# Novel dual regulatory roles of RpoA in quorum sensing regulation and social behavior switching in *Pseudomonas aeruginosa*

**DOI:** 10.1128/mbio.00032-26

**Published:** 2026-03-24

**Authors:** Huali Chen, Yonglin Liang, Xiaoqing Zhou, Wenjie Cai, Huifang Qiu, Ajai A. Dandekar, Weijun Dai

**Affiliations:** 1 Integrative Microbiology Research Center, College of Plant Protection, South China Agricultural University 12526, Guangzhou, China; 2 Zhejiang University School of Medicine & Liangzhu Laboratory, Hangzhou, China; 3 Department of Microbiology, University of Washington 7284, Seattle, Washington, USA; University of Illinois Chicago, Chicago, Illinois, USA

**Keywords:** quorum sensing, RNAP, RpoA, *Pseudomonas aeruginosa*, experimental evolution, bacterial communication

## Abstract

Understanding the social structure and evolutionary dynamics of microbial communities requires the identification and characterization of relevant mutant subpopulations. While *Pseudomonas aeruginosa* employs quorum sensing (QS) to coordinate population-wide behaviors, the social traits of many QS mutants remain poorly defined. In this study, we developed an iterative “targeted gene duplication followed by mutant screening” (TGD-MS) approach to systematically identify noncanonical QS cheater mutants. We discovered that a single-nucleotide mutation in *rpoA*, which encodes the α subunit of RNA polymerase (RNAP), produces a QS-deficient phenotype resembling QS-null mutants. This RpoA variant mutant exhibits characteristic features of social cheating, including a competitive growth advantage in mixed populations, impaired QS-dependent virulence factor production, and attenuated pathogenicity. Structural and biochemical analyses revealed that the RpoA variant impairs RNAP binding to the promoters of core QS genes (*lasI* and *lasR*), leading to diminished QS activity. Further examination of natural RpoA variants uncovered a spectrum of QS-related phenotypes, suggesting that RpoA has a dual regulatory role in QS control. Within the C-terminal domain (α-CTD) of RpoA, we identified two distinct functional determinants that, through adaptive mutations, can acquire opposing regulatory effects on QS. This enables an environmentally dependent phenotypic switch between cooperation and cheating. Our discovery of noncanonical RpoA-mediated QS cheaters expands the framework of bacterial social evolution, demonstrating that mutations outside the canonical QS circuitry can disrupt cooperative behaviors. These findings underscore how core transcriptional machinery can be evolutionarily co-opted to modulate complex social interactions in dynamic environments.

**IMPORTANCE:**

To understand how bacterial populations function and evolve, it is essential to identify socially significant subpopulations, including previously unrecognized types of cheaters. In this study, we uncover an unexpected role of RNA polymerase (RNAP) in regulating quorum sensing (QS) and QS-associated social behaviors in *P. aeruginosa*. Specifically, we demonstrate that the α subunit of RNAP (RpoA) is a key regulatory component in this process. A single-nucleotide mutation within the C-terminal domain of RpoA was found to alter QS activity, driving an environment-dependent transition between cooperative and cheating phenotypes. This discovery of this novel, noncanonical QS cheater mutant offers new insights into intra-population interactions, population stability, and evolutionary dynamics. These findings carry significant implications for microbial ecology and deepen our understanding of social evolution in bacterial communities.

## INTRODUCTION

*Pseudomonas aeruginosa* is a gram-negative opportunistic pathogen that can cause severe, life-threatening infections, such as cystic fibrosis (CF), in humans, particularly in people with compromised immune systems. This pathogen functions as a social community, exhibiting complex social behaviors that involve communicating, cooperating, and competing among individual cells (1). An interaction between subpopulation mutants significantly influences the collective behavior of the population, affecting virulence factor production, biofilm formation, antibiotic resistance development, and microbe-host interaction dynamics (2–4). Consequently, identifying and characterizing socially relevant subpopulation variant mutants are essential for understanding the social structure and evolutionary dynamics of microbial communities. However, the social traits of many *P. aeruginosa* mutants remain poorly understood. This lack of knowledge impedes our comprehension of *P. aeruginosa*’s social evolution and hinders the development of effective therapeutic strategies against infections caused by this pathogen (5, 6).

*P. aeruginosa* orchestrates its collective behaviors through a cell-cell communication system known as quorum sensing (QS), which enables the population to coordinate gene expression in a cell density-dependent manner via the production and detection of small signaling molecules (7). This pathogen possesses a complex, hierarchically organized QS network composed of three major systems: Las, Rhl, and PQS (8). The Las and Rhl systems each comprise a synthase-receptor pair—LasI/LasR and RhlI/RhlR—that produce and respond to the acyl homoserine lactones (AHLs) N-3-oxo-dodecanoyl-homoserine lactone (3OC12-HSL) and N-butanoyl-homoserine lactone (C4-HSL), respectively. These AHLs bind to their cognate transcriptional regulators, LasR and RhlR, activating transcriptional responses that regulate a wide range of genes involved in virulence, biofilm development, and metabolic adaptation (9). The third system operates through the *pqsABCDE* operon, which encodes enzymes that synthesize the quinolone signal molecule 2-heptyl-3-hydroxy-4(1H)-quinolone (*Pseudomonas quinolone* signal, PQS) (10). This molecule interacts with the transcriptional regulator PqsR (MvfR) to trigger a positive feedback loop that further amplifies PQS production (11). A critical protein encoded within this operon is PqsE, a metallo-β-hydrolase. Beyond its role in quinolone synthesis, PqsE physically interacts with RhlR, stabilizing the receptor and enhancing its affinity for target promoters (12, 13). This interaction is crucial for maximal RhlR activity, with both the canonical C4-HSL signal and PqsE binding being required for the full expression of RhlR-dependent virulence factors (14). In this QS hierarchy, the Las QS system is generally regarded as the master regulator, positioned at the top by controlling the expression of both the Rhl and PQS systems (8).

Experimental evolution approaches have demonstrated that QS social cheater mutants can emerge from populations of wild-type (WT) *P. aeruginosa*. Notably, individuals harboring mutations in *lasR* and *pqsR*, key QS regulators, were identified as social cheaters that exploit the cooperative behavior of their parental populations (15–17). RhlR mutants were not observed to arise from the wild-type strain under similar *in vitro* conditions (17). The social cheating behavior of LasR mutants was further validated in a mouse infection model (18), and these mutants have also been frequently isolated from CF patients in both acute and chronic infection stages (19–22). These findings suggest that QS-deficient mutants, particularly LasR mutants, can be positively selected in clinical environments shaped by social evolutionary pressures. In contrast, clinical isolates of *P. aeruginosa* often display a remarkable diversity of mutant subpopulations, shaped by the complex and dynamic selective pressures of the infection environment (23, 24). It is likely that among these diverse clinical populations, there exist variants with previously unrecognized social functions related to QS. Characterizing these mutants and elucidating their roles in the social and pathogenic landscape of *P. aeruginosa* could provide critical insights into infection dynamics and inform the development of novel therapeutic approaches.

Bacterial RNA polymerase (RNAP) synthesizes RNA and plays a critical role in gene transcriptional regulation (25). RNAP is a multisubunit enzyme consisting of a core subunit (α, β, β′, and ω) (26). To initiate promoter-specific transcription, the core RNAP associates with a sigma (σ) factor to form the holoenzyme, which recognizes target DNA sequences (25). Among these subunits, the α subunit encoded by *rpoA* dimerizes to create a platform for the β subunit to assemble and participates in promoter recognition (27). Structurally, the α subunit (RpoA) comprises an N-terminal domain (α-NTD) and a C-terminal domain (α-CTD), connected via a short linker (28). The α-NTD is essential for RNAP assembly (28), while the α-CTD mediates an interaction with transcriptional regulators and binds AT-rich sequences upstream of the promoter (29). RpoA is essential for cell viability and is highly conserved across diverse bacterial species (30, 31). Mutations in RNAP subunit genes have traditionally been viewed as secondary adaptive changes. Notably, mutations in *rpoA* have been shown to compensate for the fitness cost associated with rifampicin resistance, facilitating pathogen transmission (32–36). Our recent work has expanded the understanding of RpoA’s biological functions. We identified an RpoA variant mutant (T262→A, designated as RpoA262) that restores QS activity in a LasR-null background by downregulating the expression of MexEF-OprN efflux pump genes (37). This RpoA variant mutant also exhibits increased susceptibility to antibiotics that are substrates of the MexEF-OprN efflux pump (38).

To uncover novel QS-deficient cheater mutants, we recently employed a strategy termed “targeted gene duplication followed by mutant screening” (TGD-MS) (37). Using this approach, we identified GroEL mutants as non-LasR QS cheaters (accompanying manuscript). In this study, we applied TGD-MS iteratively, engineering *P. aeruginosa* PAO1 with additional copies of *lasR* and *groEL* and subjecting it to experimental evolution. This led to the discovery of a new RpoA mutant (L289→S, hereafter RpoA289) that affects QS-regulated proteolysis. In contrast to the QS-enhancing RpoA262 variant mutant (37), the RpoA289 variant mutant exhibited QS-deficient phenotypes, including reduced transcription of QS regulatory genes and decreased production of signaling molecules. Furthermore, this RpoA289 mutant showed attenuated virulence in mammalian host cells, likely due to the diminished production of QS-controlled virulence factors. Functionally, the QS-deficient RpoA289 mutant acts as a social cheater, gaining a fitness advantage by exploiting public goods produced by QS-proficient cells. Structural modeling suggested that the RpoA variant alters the three-dimensional conformation of the α-CTD, reducing RNAP binding affinity to QS gene promoters (*lasI* and *lasR*), thereby modulating QS activity. Additionally, we found that *rpoA* mutations are prevalent among natural *P. aeruginosa* isolates, with these variant mutants exhibiting diverse QS regulatory effects. Based on these observations, we proposed that two distinct functional determinants, topologically separated within the α-CTD, mediate opposing effects on QS regulation. In summary, our study demonstrates the utility of iterative TGD-MS for uncovering hidden regulatory components in complex pathways. We reveal a dual role for RpoA in QS regulation, expanding its known biological functions. Furthermore, we show that mutations in *rpoA* can drive divergent social behaviors in bacterial populations, enabling transitions between cooperative and cheating strategies.

## RESULTS

### Iterative application of the TGD-MS approach to identify noncanonical QS cheater mutants

**Fig 1:**
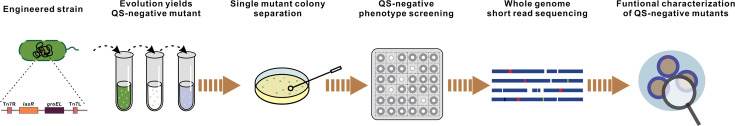
Schematic overview of the iterative “targeted gene duplication followed by mutant screening” (TGD-MS) approach. The illustration depicts the iterative TGD-MS strategy developed in this study, which aims to identify mutations in genes other than *lasR* and *groEL*. The evolution experiment begins with a recombinant PAO1 strain carrying single additional copies of *lasR and groEL*. The bacteria are spread on skim milk agar to identify quorum sensing (QS)-inactive mutants that fail to secrete QS-dependent proteases.

In our recent study, we utilized a TGD-MS approach to identify *Pseudomonas aeruginosa* QS cheater mutants involving GroEL (accompanying manuscript). To expand the discovery of unknown noncanonical QS cheaters in *P. aeruginosa*, we iteratively applied the TGD-MS strategy (Fig. 1). We began by engineering the *P. aeruginosa* strain PAO1 used for *in vitro* QS evolution. Since GroEL mutants were previously identified using a recombinant *P. aeruginosa* PAO1 strain harboring two copies of *lasR* (accompanying manuscript), we further integrated an additional wild-type copy of *groEL* into a neutral site of this strain. The resulting engineered strain contained dual copies of both *lasR* and *groEL*. We reasoned that during experimental evolution, spontaneous mutations in either *lasR* or *groEL* would be compensated by the other intact copy, preventing mutant strains from gaining a fitness advantage. This selective suppression of known LasR and GroEL QS cheater mutants would thereby promote the emergence and enrichment of other cheater mutants within the evolving population. This strategy provides a systematic way to explore previously unrecognized contributors to QS-mediated cooperation and competition.

### Protease-negative mutants can emerge from the engineered strain

**Fig 2:**
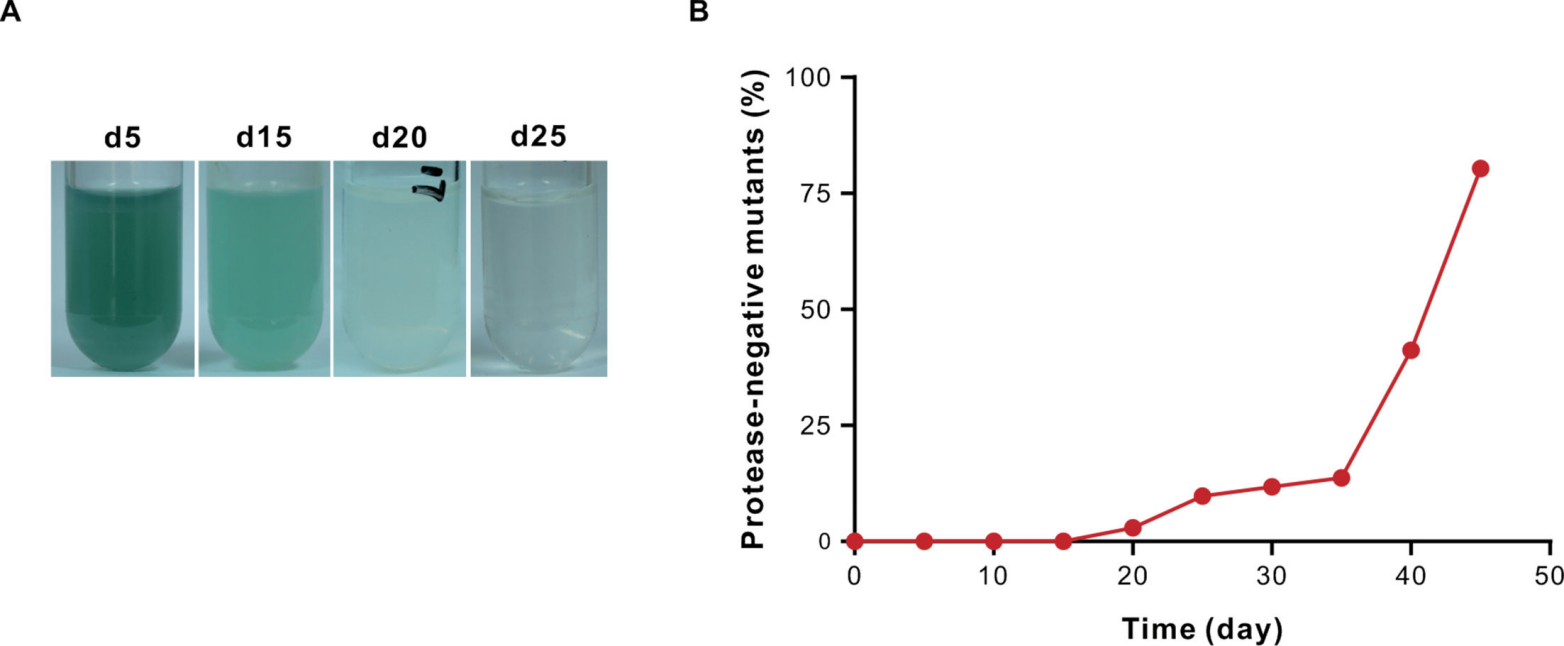
Identification of quorum sensing (QS)-inactive mutants from a recombinant PAO1 strain with an extra copy of *lasR* and *groEL*. (**A**) Casein medium cultures of the recombinant PAO1 strain carrying an extra copy of *lasR* and *groEL* photographed over time. The culture is more translucent by day 20, indicating the emergence of QS-inactive mutants. (**B**) The frequency of protease-negative colonies obtained from three iterative “targeted gene duplication followed by mutant screening” (TGD-MS) experiments. QS-inactive colonies were identified by their lack of protease activity on skim milk agar. Data are shown as means ± SD (*n* = 3).

The engineered strain (dual *lasR/groEL*) was then subjected to experimental evolution in the casein medium, where bacterial growth is dependent on QS-regulated extracellular proteases that hydrolyze casein to release utilizable carbon and energy (15, 16). Using skim milk agar plate assay to monitor QS activity (protease-catalyzed clearance zones), we observed protease-negative mutants emerging by day 20 (Fig. 2). Reduced pigment production and cell culture clarification coincided with the rise of these mutants, suggesting mutations leading to QS-deficient phenotypes. As extracellular proteases are public goods benefiting the population (15, 16), these mutants we identified are likely social cheaters that exploit protease-producing cooperators.

### Mutations in *rpoA* underline the QS-dependent proteolytic defect

To identify the genetic determinants of the observed protease-deficient phenotype, we first verified the integrity of *lasR* and *groEL* in the mutant clones. Both genes retained wild-type sequences, indicating that other mutations were responsible for the observed phenotype. Whole-genome re-sequencing (WGS) of protease-negative clones (Table S1 and Fig. S1) revealed a single-nucleotide mutation (nucleotide A4754559→G, amino acid L289→S) in *rpoA* and several mutations in noncoding regions (Table S2). The *rpoA* gene encodes the α-subunit of RNAP, a critical transcriptional regulator in *P. aeruginosa* (37, 39). To validate the functional impact of this mutation, we complemented the evolved mutant with a wild-type copy of *rpoA*. This complementation restored protease activity, as evidenced by the reappearance of clearance zones on the skim milk agar (Fig. S2). Similarly, an engineered isogenic *rpoA* mutant (referred to as RpoA289) generated in the wild-type background displayed a comparably suppressed proteolytic activity. Furthermore, introducing an episomal copy of wild-type *rpoA* into this mutant rescued its protease function. Together, these findings demonstrate that the mutations in *rpoA* can suppress QS-dependent protease activity in both evolved isolates (bearing dual *lasR/groEL*) and engineered mutants.

### A dual regulatory role of RpoA in QS regulation

**Fig 3:**
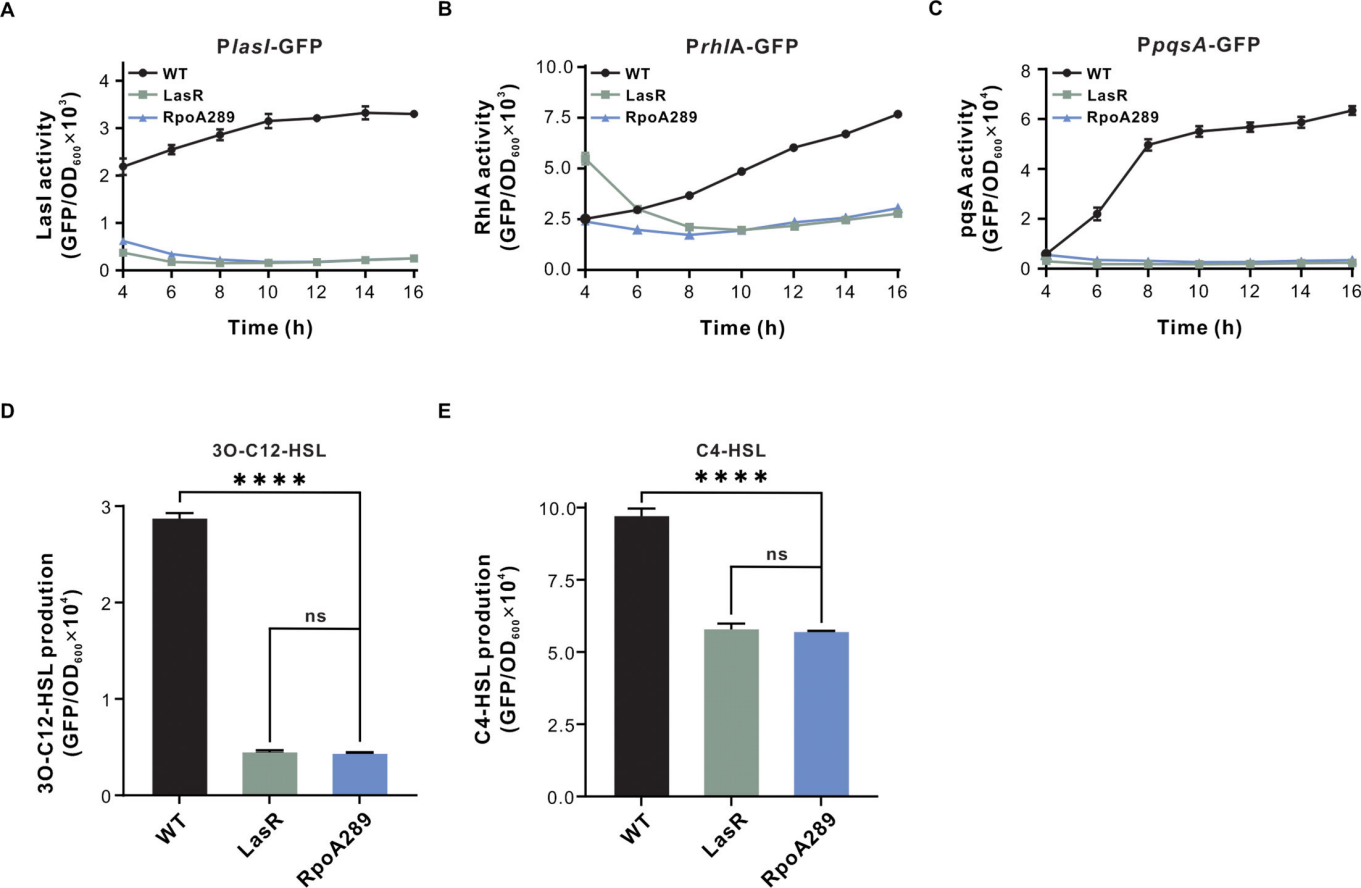
The RpoA289 mutant exhibits reduced quorum sensing (QS)-related activities. (**A–C**) Las- (**A**), Rhl- (**B**), and PQS-responsive (**C**) QS activities in the indicated strains. These activities were measured using the reporters P*lasI*-GFP (Las system), P*rhlΑ-*GFP (Rhl system), and P*pqsA*-GFP (PQS system), respectively. Fluorescence values, expressed as relative fluorescence units (RFU) normalized to OD_600_, were quantified after 18 h of bacterial growth (*n* = 4). (**D and E**) Relative concentrations of the QS signal molecules 3OC12-HSL (**D**) and C4-HSL (**E**) in the tested strains. Data are presented as means ± standard deviation (SD) (*n* = 6). A one-way analysis of variance (ANOVA) with Bonferroni posttest was used for statistical analysis (*P < 0.05, **P < 0.01, and ***P < 0.001). WT, wild-type PAO1 strain; LasR, LasR-null mutant; RpoA289, the RpoA289 variant mutant.

To investigate the impacts of the RpoA289 mutant on QS circuits, we analyzed QS activity using GFP-based reporter systems in relevant *P. aeruginosa* strains. These reporters were constructed by fusing the promoter region of key QS-regulated genes (*lasI*, *rhlA*, and *pqsA*) upstream of the *gfp* gene, resulting in P*lasI*-GFP, P*rhlA*-GFP, and P*pqsA*-GFP, respectively. These reporters reflect the transcriptional activities of Las, Rhl, and PQS QS circuits, respectively. In agreement with the observed loss of proteolysis (Fig. 2; Fig. S2), the RpoA289 mutant exhibited a markedly reduced fluorescence from all three QS reporters, comparable to those detected in the QS-deficient LasR-null mutant (Fig. 3A through C). This finding suggests that the Las, Rhl, and PQS QS circuits were all suppressed in the RpoA289 mutant. Production of the QS signals 3OC12-HSL and C4-HSL was also significantly decreased in the RpoA289 mutant relative to the wild-type strain (Fig. 3D and E). Together, these results indicate that the RpoA289 variant negatively regulates QS, downregulating the Las, Rhl, and PQS QS circuits. By contrast, another RpoA262 mutant (nucleotide T4754641→C, amino acid T262→A) acted as a QS-positive regulator, enhancing QS-dependent phenotypes, as reported in our recent study (37). Taken together, our findings reveal a dual regulatory role of RpoA in QS regulation, wherein distinct mutations in *rpoA* can either repress or enhance QS activity, depending on the specific amino acid substitution.

### The RpoA289 mutant can function as a QS cheater

**Fig 4:**
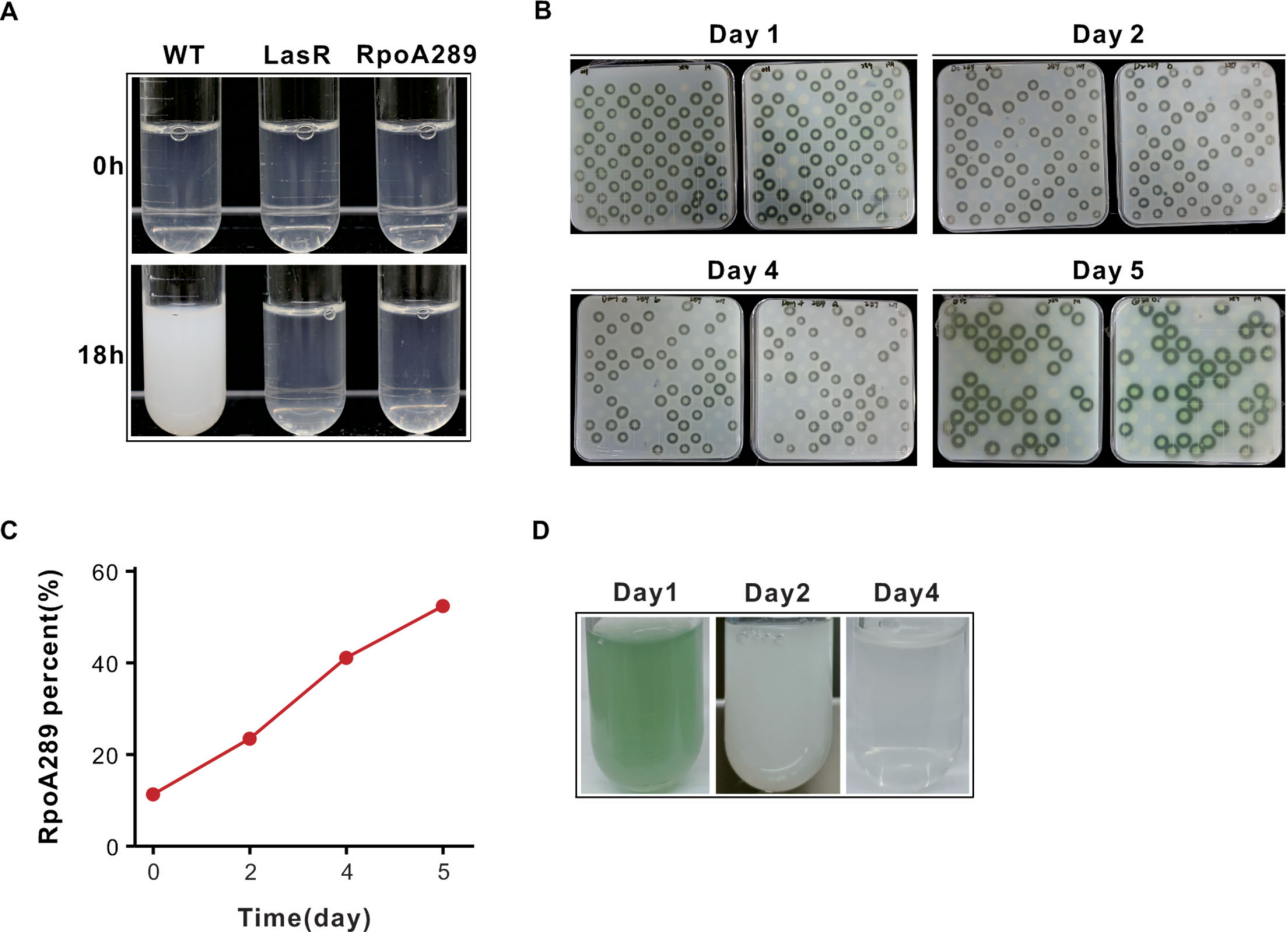
The RpoA289 mutant demonstrates a competitive advantage over the wild-type strain. (**A**) Monoculture growth of the indicated strains in casein medium. Culture tubes were photographed after 18 h of incubation. (**B–D**) Competitive fitness of the RpoA289 mutant against wild-type strain PAO1. (**B**) Co-cultures were initiated at 90:10 (mutant: wild-type) ratio in casein medium, and bacterial populations were quantified using skim milk agar plating after 24 h. (**C**) The relative frequency of the RpoA289 mutant over time. (**D**) Representative images of co-cultures at specified time points. Experiments were performed with three independent biological replicates, each repeated at least three times, with consistent results.

We next investigated whether the QS-deficient phenotypes of the RpoA289 mutant affect its social behavior in a population context. Its emergence and persistence in the evolving population (Fig. 2) suggest it may have a selective advantage over the wild-type. These observations imply that the RpoA289 mutant may possess cheating traits, like LasR-null mutants that exploit communal resources without contributing to their production. To test this hypothesis, we examined the growth of the RpoA289 mutant in monoculture and co-culture with the wild-type strain in casein medium, which requires QS-dependent protease production for nutrient acquisition (15). In monoculture, both LasR-null and the RpoA289 mutants could not grow in casein medium, presumably due to deficient protease production (Fig. 4A). However, when co-cultured with the wild-type strain, the percentage of the RpoA mutant cells increased steadily over time (Fig. 4B and C), indicating a fitness benefit derived from exploiting proteases produced by the wild-type partner. Notably, the rise in RpoA289 abundance was accompanied by a reduction in the total population yield, as evidenced by decreased turbidity and diminished pigment production in co-culture (Fig. 4D). We reasoned that the mutant’s proliferation imposes a burden on the community, potentially leading to population collapse due to overexploitation of shared resources. Based on these findings, we concluded that the RpoA289 mutant is an atypical QS cheater in evolving *P. aeruginosa* populations.

### RpoA289 reduces bacterial pathogenicity

**Fig 5:**
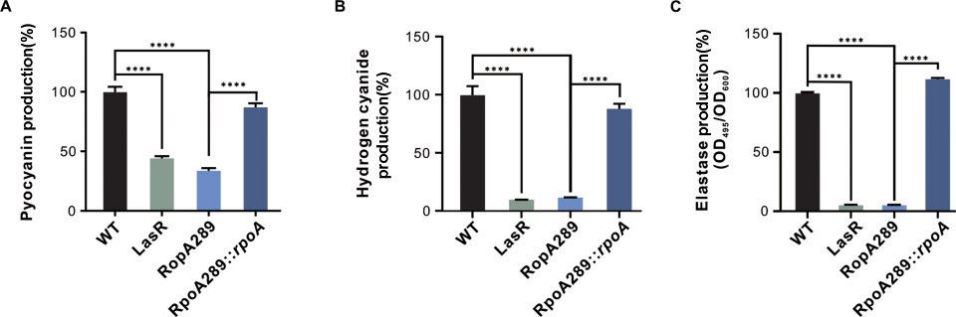
Production of quorum sensing (QS)-controlled virulence factors in the RpoA289 mutant. (**A**) Pyocyanin production (OD_695_/OD_600_ values) in the indicated strains. (**B**) Cyanide production in various strains, normalized to wild-type PAO1 for comparison. (**C**) Elastase activity (OD_495_/OD_600_) in the tested strains. Data presented means ± standard deviation (SD) from three independent experiments (*n* = 3). Statistical significance was assessed using a one-way analysis of variance (ANOVA), followed by Bonferroni’s *post hoc* test for multiple comparisons (*P < 0.05, **P < 0.01, and ***P < 0.001). WT, wild-type PAO1 strain; LasR, LasR-null mutant; RpoA289, the RpoA289 variant mutant; RpoA289::*rpoA*, the RpoA289 variant mutant integrated with a miniTn7-P*rrnB-rpoA* construct.

Given that QS tightly controls the production of key virulence factors in *P. aeruginosa* (7), we asked whether the RpoA289 mutant affects bacterial pathogenicity. To address this question, we first examined the production of three QS-regulated virulence factors—pyocyanin, hydrogen cyanide (HCN), and elastase—in both the constructed isogenic RpoA289 mutant and an independently isolated mutant clone carrying the same *rpoA* mutation. As expected, all three factors were markedly reduced compared to the wild-type strain (Fig. 5A through C). Complementation with a wild-type copy of *rpoA* restored virulence factor production, confirming that the QS-deficient phenotypes were due to the RpoA289 mutation. To determine if RpoA on virulence operates depending on intact QS circuitry, we generated a double mutant with the *rpoA* variant and lacking *lasR* (LasR-RpoA289). We found that the virulence factor profile of this double mutant closely resembled that of the single LasR-null mutant (Fig. S3). Furthermore, complementation of the LasR-null mutant with wild-type *rpoA* (LasR::*rpoA*) did not restore virulence factor production. These results indicate that the impact of RpoA on pathogenicity is dependent on functional LasR.

**Fig 6:**
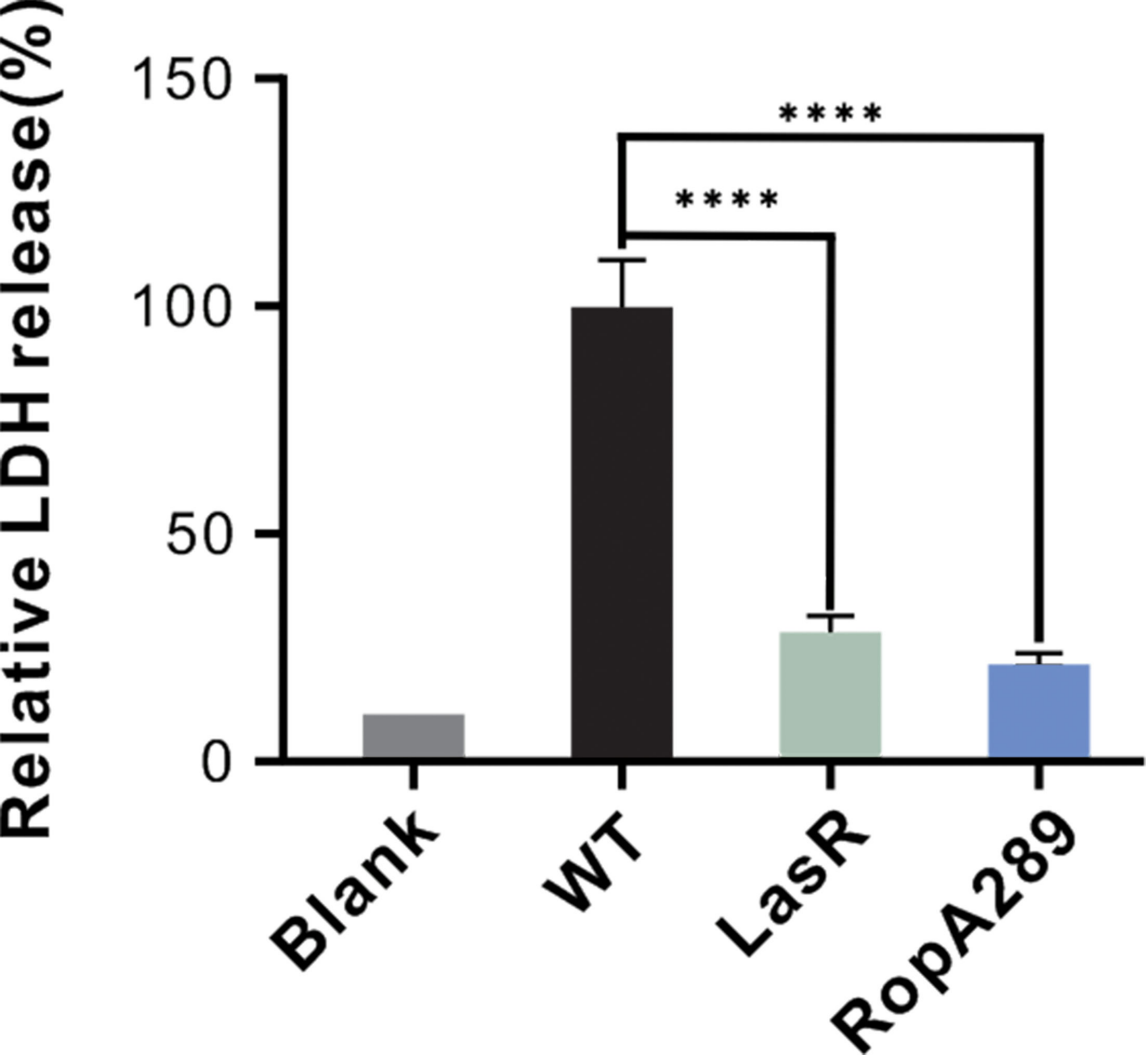
The RpoA289 mutant exhibits significantly reduced cytotoxicity toward eukaryotic cells. A cytotoxicity assay was conducted using Chinese hamster ovary (CHO) cells infected with equal amounts of the indicated bacterial strains at a starting multiplicity of infection (MOI) ratio of 5: 1. After incubation for 6 h, lactate dehydrogenase (LDH) released from the cytosol of infected cells was measured as an indicator of cell lysis. The LDH release observed with the wild-type (WT) strain was normalized to 100% for comparison. Data are presented as means ± standard deviation (SD) (*n* = 3). A one-way analysis of variance (ANOVA) with a Bonferroni *post hoc* test was applied (*P < 0.05, **P < 0.01, and ***P < 0.001).

To assess the functional consequences of this reduction in virulence, we evaluated the cytotoxicity of the RpoA289 mutant toward mammalian cells using a lactate dehydrogenase (LDH) release assay in Chinese hamster ovary (CHO) cells. Consistent with its attenuated virulence factor profile, the RpoA289 mutant induced significantly lower cytotoxicity than the wild-type, comparable to that of a LasR-null mutant (Fig. 6). Taken together, these results demonstrate that the emergence and invasion of the QS-deficient RpoA289 mutant not only confer a selective advantage through cheating behavior but also reduce the overall bacterial pathogenicity of the whole *P. aeruginosa* population by impairing QS-regulated virulence factors.

### Amino acid substitution in the RpoA289 variant induces structural changes

To begin to understand the molecular basis of the alteration in QS regulation observed in the RpoA289 mutant, we investigated whether the amino acid substitution in the RpoA289 variant results in detectable modification in its three-dimensional structure. We first compared the predicted secondary structures of the wild-type RpoA protein and the RpoA289 variant. The analysis revealed differences in the proportion of helices, strands, and coils between the two proteins (Table S3), suggesting that the mutation may influence the overall fold and structure. We then used AlphaFold3 (40) to model the three-dimensional structure of both proteins. Structural alignment of the predicted models revealed a modest overall difference (RMSD = 1.414), with the α-NTD region appearing largely conserved while notable structure differences were observed in the α-CTD region (Fig. S4), in which the amino acid substitution is localized. These findings indicate that the single-amino acid substitution in the RpoA289 variant induces a local structural alteration in the α-CTD region.

### The RpoA289 variant attenuates RNAP binding to the *lasI* and *lasR* promoters

**Fig 7:**
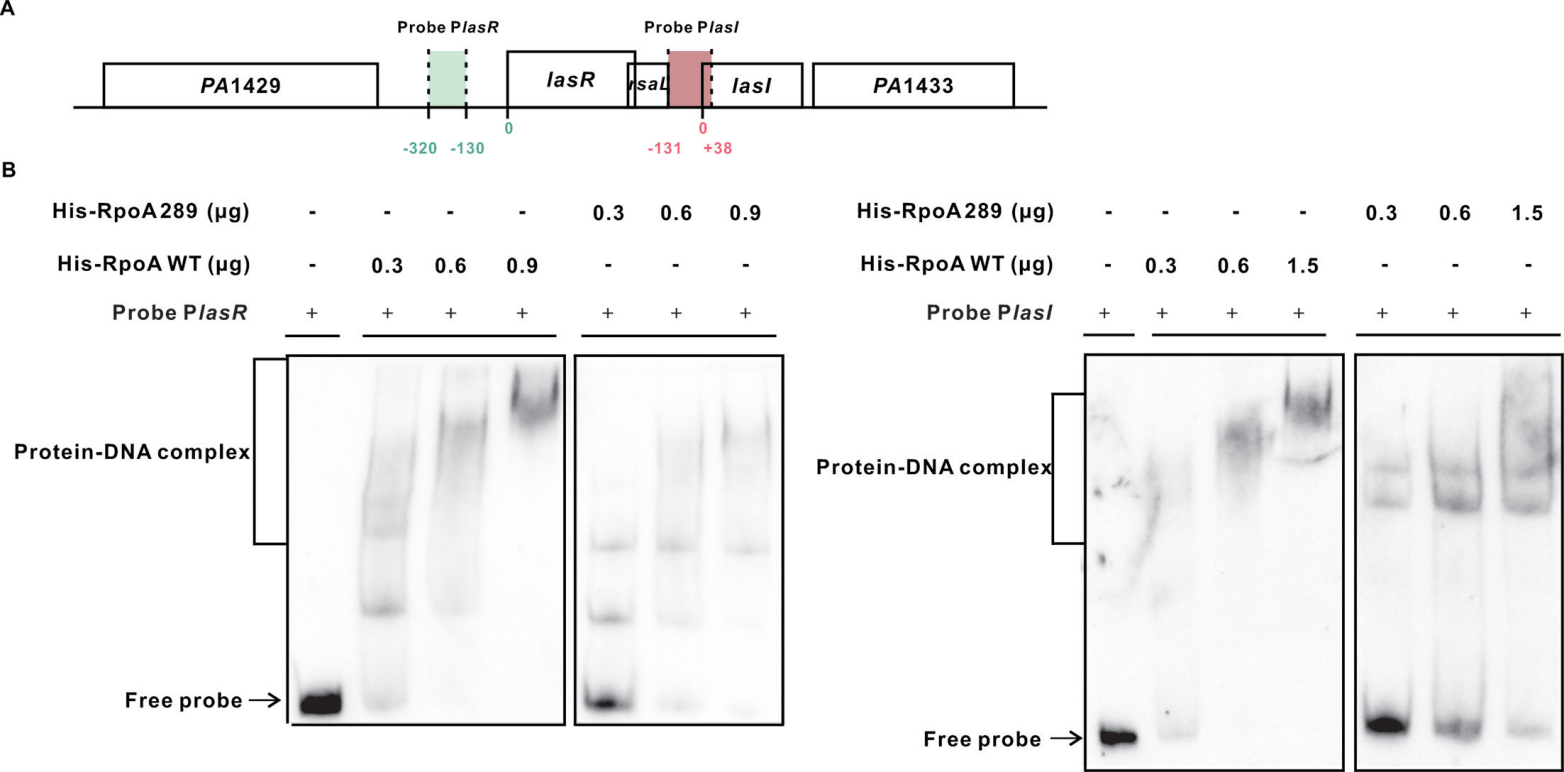
RpoA289 impairs RNA polymerase (RNAP) binding to target promoters. (**A**) Schematic representation of the DNA probe used in electrophoretic mobility shift assay (EMSA) with recombinant wild-type (WT) RpoA or the RpoA289 variant. (**B**) EMSA analysis reveals that RNAP containing WT RpoA robustly binds the *lasI* and *lasR* promoter probes, forming stable DNA-protein complexes. In contrast, RNAP incorporating the RpoA289 variant exhibits significantly reduced binding affinity, as indicated by fainter band intensity and slower migration of the DNA-protein complexes. Free probe DNA is visible at the bottom of the blot.

As RpoA is a core component of RNAP and its α-CTD is critically involved in promoter recognition and transcription initiation (25), we hypothesized that the structural changes in RpoA289 α-CTD could impair RNAP’s ability to bind to QS gene promoters, thereby reducing transcriptional activity. Because the Las QS system sits atop a hierarchy, modulating Rhl and PQS QS (7), we focused our analysis on the transcriptional regulation of *lasI* and *lasR* in the RpoA289 mutant. A reporter assay revealed significantly reduced transcription of both *lasI* and *lasR* in the RpoA289 mutant compared to the wild-type (Fig. S5). This observation suggested that the mutated RpoA289 protein may compromise RNAP-promoter interactions. To directly assess this possibility, we performed an electrophoretic mobility shift assay (EMSA). In this EMSA, His-tagged versions of wild-type and mutant RpoA289 proteins were expressed in *P. aeruginosa* PAO1. The corresponding RNAPs were purified and incubated with DNA probes containing the *lasI* and *lasR* promoter regions. EMSA revealed a reduced binding affinity of RNAP complexes containing the RpoA289 variant, as evidenced by weaker or delayed mobility shifts relative to those with wild-type RpoA (Fig. 7). These findings provide insights into how structural modifications in the α-CTD of RpoA289 lead to impaired RNAP-DNA interactions, thereby reducing the transcription of Las QS system genes and dampening the activity of the entire QS network.

### RpoA variant mutants from natural environments display diverse QS-controlled phenotypes

**Fig 8:**
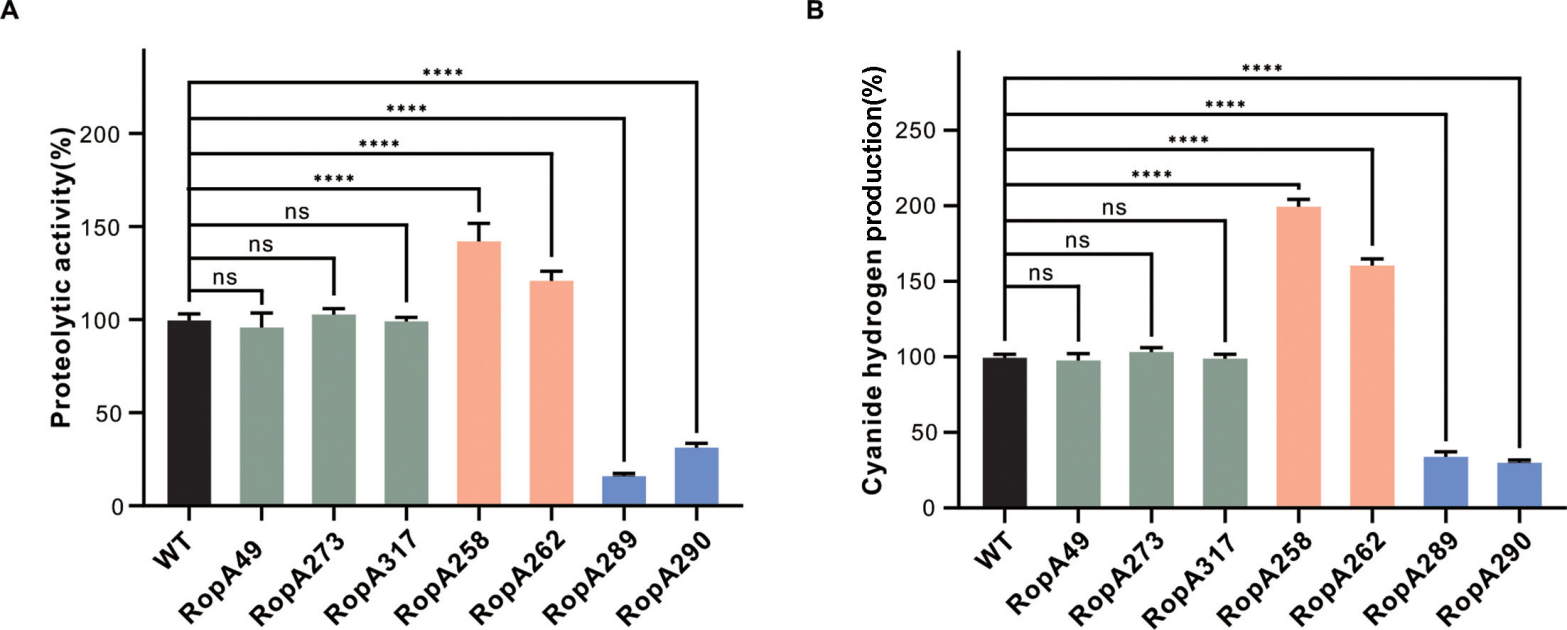
Quorum sensing (QS)-controlled phenotypes in RpoA variant strains. The impacts of RpoA variants on QS-controlled phenotypes were assessed by comparing proteolytic activity and hydrogen cyanide production across different variant strains. (**A**) Proteolytic activity was measured using a skim milk agar plate assay, where protease secretion was quantified based on the size of hydrolysis zones around bacterial colonies. (**B**) Production of hydrogen cyanide was determined by incubating strains in 6-well plates at 37°C for 24 h, followed by analysis using cyanide-sensitive filter papers. Wild-type PAO1 cyanide levels were set as 100% for normalization. WT, wild-type PAO1 strain, RpoA49 variant (S49→F), RpoA258 (D258→G), RpoA262 (T262→A), RpoA286 (V286→M), RpoA289 (L289→S), RpoA290 (K290→N), and RpoA319 (N319→S). Data represent the means ± standard deviation (SD) (*n* = 3). Statistical comparisons among groups were performed using a one-way analysis of variance (ANOVA) with Bonferroni correction for multiple comparisons (*P < 0.05, **P < 0.01, and ***P < 0.001).

To explore the occurrence of RpoA289-like variants in natural environments, we searched the *Pseudomonas* database using the BLAST algorithm with the wild-type PAO1 RpoA protein sequence (NP_252928.1) as the query. This search identified 157 RpoA variant sequences with single-amino acid substitution from *P. aeruginosa* isolates originating from various environmental sources (Table S4). Notably, the RpoA289 variant appeared 6 times in the data set (Fig. S6), indicating that strains harboring this mutation may possess adaptive advantages in both experimental and natural settings. To assess the functional impacts of naturally occurring RpoA variants on the QS circuits, we constructed isogenic PAO1 mutants each carrying a single-amino acid substitution corresponding to those found in environmental isolates. We then evaluated their QS-regulated phenotypes, including extracellular protease and hydrogen cyanide production. Based on these phenotypic assays, the RpoA variants were classified into three groups: group I (unchanged QS activity; RpoA49, RpoA273, and RpoA317), group II (up-regulated QS activity; RpoA258 and RpoA262), and group III (downregulated QS activity; RpoA289 and RpoA290) (Fig. 8; Fig. S7). Interestingly, the RpoA258 variant (amino acid D258→G) exhibited QS-enhancing effects similar to those seen in the RpoA262 variant, while the RpoA290 variant (amino acid D290→N) mirrored the QS-repressing phenotype of RpoA289. These findings suggest that *rpoA* is a hotspot for functionally significant mutations. Together, our data reveal that naturally selected RpoA variants play diverse regulatory roles in QS networks, leading to distinct phenotypes.

### Two distinct functional determinants in the α-CTD of RpoA

**Fig 9:**
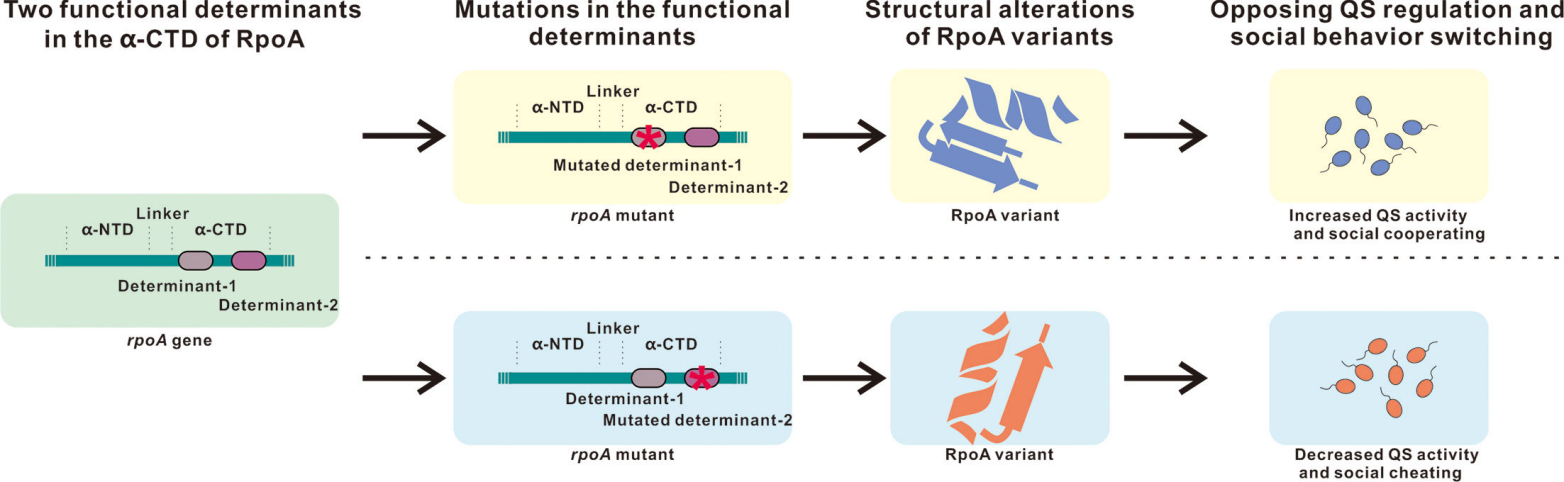
Schematic representation of distinct quorum sensing (QS) regulatory mechanisms and social behaviors mediated by RpoA α-CTD variants. Mutations in the two functional determinants of RpoA induce distinct three-dimensional structural alterations. These conformational changes differentially modulate QS activity, ultimately driving a phenotypic switch between cooperative and cheating behaviors in bacterial populations. Specifically, certain variants enhance QS-mediated cooperation by promoting signal production or response, while others favor cheating by exploiting public goods without contributing to their synthesis.

To understand the structural and functional basis of the observed phenotypic diversity, we mapped the amino acid substitutions of the functionally characterized RpoA variants onto a sequence alignment of RpoA homologs from various bacterial species (Fig. S8). This analysis revealed that all substitutions occurred at less conserved positions, suggesting that they do not impair the essential functions of RpoA. Intriguingly, the substitutions associated with altered QS activity—RpoA258, RpoA262, RpoA289, and RpoA290—were all located within the α-CTD region of RpoA. These QS-associated mutations form two spatially distinct clusters within the α-CTD: one involving residues D258 and T262 (linked to QS activation) and the other involving adjacent residues L289 and D290 (associated with QS repression). Structural modeling based on the predicted 3D conformation of RpoA confirmed that each pair of substitution clusters is closely positioned in three-dimensional space but is topologically separated from the other pair (Fig. S9). These spatially distinct clusters represent two independent functional determinants within the α-CTD that oppositely regulate QS. We propose that these dual-functional determinants enable *P. aeruginosa* to flexibly adapt its QS-regulated social behaviors in response to environmental changes, thereby maintaining evolutionary fitness in dynamic populations (Fig. 9).

## DISCUSSION

In this study, we identified a novel noncanonical QS cheater mutant in *P. aeruginosa*, characterized by a single-nucleotide substitution in the *rpoA* gene, which encodes the RNA polymerase α-subunit. This mutant, RpoA289, exhibited classic features of social cheating under conditions that require bacterial cooperation. Unlike previously described QS cheater mutants, which carry mutations in QS signal receptor genes and are therefore blind to QS signals (15–17), the RpoA289 mutant does not carry mutations in any known QS regulatory loci. Notably, although the RpoA289 mutant retains an intact QS gene circuit, it exhibited drastically reduced expression of both *lasI*, the synthase for the 3OC12-HSL signal molecule, and *lasR*, the QS master regulator. As a result, it mimics both LasI- and LasR-null mutants, functioning as both QS signal-negative and QS signal-blind. The discovery of the RpoA289 mutant expands the conceptual framework of bacterial social evolution, highlighting that mutations outside the QS circuit—particularly in global transcriptional regulators like RpoA—can disrupt QS-dependent cooperation. This finding reveals the complexity and vulnerability of QS regulatory networks, where perturbations in global transcriptional regulators such as RpoA can generate social cheaters that bypass canonical QS surveillance mechanisms.

Our identification of RpoA289 and the GroEL mutant (accompanying manuscript) underscores the existence of noncanonical cheaters carrying mutations outside known QS regulatory genes. This work was facilitated by our iterative use of the TGD-MS strategy, which suppressed more likely mutations such as those in *lasR* and *groEL*. Our approach revealed an RpoA mutant that could not be discovered using traditional genetic tools, such as transposon-insertion mutagenesis, since RpoA is essential for bacterial viability (30). This result highlights the value of iterative TGD-MS as a general strategy for probing regulatory networks in diverse biological systems. We propose that, with appropriate selection pressures, this approach can be adapted to explore unidentified regulatory elements in a broad range of organisms and pathways. By suppressing the emergence of mutations that are more likely in the conditions of our experiments, we show that the TGD-MS approach allowed us to interrogate, in an unbiased way, a naturally occurring variant of *rpoA*.

Previous studies have shown that mutations in RNAP subunit genes can facilitate bacterial adaptation to environmental stressors. For instance, mutations in the RNAP β subunit gene *rpoB* confer resistance to rifampicin (41), but often incur a fitness cost (36, 42–44). This cost can be mitigated by compensatory mutations in *rpoA*, *rpoB*, and *rpoC*, which influence subunit interactions between the RNAP complex (32–36). Beyond fitness compensation, our recent study shows that RNAP mutations may also influence regulatory circuits such as QS. A spontaneous point mutation in *rpoA* (T262A or RpoA262) was found to restore QS activity in a LasR-null background by downregulating the *mexEF-oprN* efflux pump operon, thus positively modulating QS (37). In contrast, our current study identifies RpoA289 as a negative regulator of the QS circuit. This RpoA289 mutant displays QS-inactive phenotypes similar to LasR-null mutants and behaves as a social cheater within populations. Moreover, we identified additional RpoA variants (RpoA258 and RpoA290) from natural isolates that exert opposing effects on QS regulation, suggesting functional diversification and environmental selection for these variants. Overall, our findings reveal that the *rpoA* gene can acquire distinct, even opposing, regulatory functions through specific adaptive mutations. This functional plasticity enables *P. aeruginosa* to modulate the production of QS-regulated virulence products, thereby facilitating adaptation to diverse and fluctuating environments. These results underscore the pivotal role of global transcriptional regulators like RpoA in mediating evolutionary adaptation. More broadly, the discovery of RpoA’s dual influence on QS regulation significantly expands the known functional repertoire of bacterial RNA polymerase subunits and highlights their potential involvement in shaping bacterial social behavior in dynamic ecological contexts.

Our present study reveals that RpoA mutants are relatively common in isolates from environmental sources, with the number of RpoA α-CTD mutants exceeding that of α-NTD mutants (Fig. S4). The prevalence of RpoA α-CTD mutants suggests a potential selective advantage, possibly due to their roles in modulating transcriptional responses critical for bacterial adaptation to diverse environmental stresses. Among these identified RpoA α-CTD mutants, we predict that some may exert regulatory impacts on the QS circuit similar to the RpoA289 mutant, such as the RpoA290 mutant. Specific point mutations in the *rpoA* gene differentially regulate the QS circuit: RpoA289 and RpoA290 mutants exhibited QS-negative activity, whereas RpoA258 and RpoA262 mutants displayed QS-positive phenotypes (Fig. 9; Fig. S5). These results highlight that even within the same gene, localized mutations can exert opposing regulatory effects on QS, underscoring the nuanced role of RNAP in bacterial cell-to-cell communication. Structurally, the RpoA consists an N-terminal domain (α-NTD), a short linker, and a C-terminal domain (α-CTD) (27). The α-NTD is essential for RNAP assembly, while the α-CTD mediates an interaction with transcriptional factors and promoter DNA (29). Consistent with this functional division, the RpoA49 mutant—bearing an amino acid substitution in the α-NTD—has no detectable effects on QS. Notably, this mutant was previously reported to be associated with fitness cost by influencing RNAP subunit interactions (32). By contrast, all QS-modulating RpoA mutants identified in this study harbor substitutions exclusively within the α-CTD, highlighting the functional importance of this domain in QS regulation. These mutations cluster into at least two functionally distinct regions (residues 289/290 and 258/262), suggesting that single-amino acid changes in these determinants induce distinct structural rearrangements. Such conformational alterations could further influence interactions between the RpoA α-CTD and transcriptional regulators, including Vfr, RpoS, and QS regulators like LasR and RhlR, as well as modify the RNAP binding affinity for promoter elements. These combined effects may ultimately contribute to the distinct QS regulatory outcomes observed in RpoA variants. These findings provide new mechanistic insights into how RNAP fine-tunes QS circuits through specific structural changes in the α-CTD, offering potential targets for modulating bacterial communication in therapeutic and biotechnological applications.

In this study, we demonstrate that RNAP, through its α subunit RpoA, also plays a critical role in regulating QS and QS-associated social behaviors in *P. aeruginosa*. This indicates that RNAP not only governs intracellular gene regulation but also influences population-level social dynamics. Thus, RNAP controls social behavior, flexibly coordinating QS-mediated interactions in response to changing environmental conditions. Our findings significantly broaden the known functions of bacterial transcription machinery by revealing their influences beyond core transcription, extending to the regulation of social behaviors that underpin bacterial community organization. Moreover, these insights contribute to microbial sociobiology, suggesting that fundamental transcriptional components can be repurposed through evolution to mediate complex intercellular interactions in fluctuating environments.

## MATERIALS AND METHODS

### Bacterial strains and growth conditions

*P. aeruginosa* PAO1 (45) and mutant derivatives were grown in Luria Bertani (LB) broth (containing 10 mg/mL tryptone, 5 mg/mL yeast extract, and 10 mg/mL NaCl) at 37°C. LB broth was buffered with 50 mM 3-(N-morpholino) propanesulfonic acid, pH 7.0 (LB-Mops broth). M9 minimal media (15, 46) supplemented with 1% sodium caseinate (C8654, Sigma-Aldrich, New Zealand) (M9-1% casein medium) or 0.5% casamino acids (A100851, Sangon Biotech, Shanghai, China) (M9-0.5% CAA) as the sole carbon source was used for evolution experiments at 37°C. Unless otherwise specified, *P. aeruginosa* strains were cultured in 14-mL Falcon tubes (Corning, Shanghai, China) containing 3 mL medium, with shaking (250 rpm) at 37°C. *Escherichia coli* was grown in LB broth at 37°C. Details on bacterial strains and plasmids used in this study are shown in Table S5.

### Construction of *P. aeruginosa* mutants

Site-directed mutants (RpoA49, RpoA258, RpoA262, RpoA273, RpoA289, RpoA290, and RpoA317) were constructed in *P. aeruginosa* PAO1 using a well-established homologous recombination protocol (47). For each mutation, 500–1,000 bp flanking regions surrounding the targeted nucleotide substitution or the complete gene sequence were PCR-amplified and cloned into the gentamicin-resistant pEXG2 suicide vector (45, 47) using the Vazyme ClonExpress II One Step Cloning Kit (Vazyme Biotech, Nanjing, China). Recombinant plasmids were introduced into PAO1 via triparental mating utilizing *E. coli* HB101/pRK2013 as a conjugation helper (48). Primary selection was performed on *Pseudomonas* isolation agar (PIA; containing 20 g/L peptic digest, 1.4 g/L MgCl₂, 10 g/L K₂SO₄, 0.025 g/L triclosan, and 13.6 g/L agar) supplemented with 100 μg/mL gentamicin. Secondary selection for plasmid excision was conducted on LB agar containing 10% (wt/vol) sucrose. All mutations were verified through PCR amplification followed by Sanger sequencing, with primers detailed in Table S6.

### Complementation of the RpoA289 mutant

The *rpoA* gene was cloned into the arabinose-inducible pJN105 expression vector using the Vazyme ClonExpress II One Step Cloning Kit, yielding plasmid pJN105-rpoA. Both the recombinant plasmid and empty pJN105 vector (negative control) were introduced into the PAO1 RpoA289 mutant strain via triparental mating. Transconjugants were cultured in LB-Mops broth at 37°C under aerobic conditions. To assess complementation, *rpoA* expression was induced with 1 mM L-arabinose, followed by bacterial harvest after 20 h of incubation for subsequent analysis.

### Evolution experiments

Three biological replicates of *P. aeruginosa* PAO1, each harboring a miniTn7-GroEL-RpoA construct integrated at the *att*Tn7 genomic locus, were independently inoculated into 3 mL of LB-Mops broth and grown overnight. For the evolution experiment, 30 μL aliquots of each culture were transferred into 3 mL of M9 minimal medium supplemented with 1% casein in 14-mL Falcon tubes (Corning, Shanghai, China). Daily passaging was performed by serial transfer of 30 μL bacterial suspension into fresh M9-casein medium, maintaining this regime for 45 consecutive days. To monitor population dynamics, samples were plated on LB agar every fifth day. Following the evolution period, colonies were assessed for extracellular protease activity using the skim milk clearance assay to evaluate phenotypic changes.

### Skim milk protease assay

The extracellular proteolytic activity of *P. aeruginosa* strains was assessed using skim milk agar plates, where bacterial protease secretion generates a visible zone of clearance around colonies. Overnight cultures were first grown on LB agar, and individual colonies were then spot-inoculated onto skim milk agar plates (composed of 25% [vol/vol] LB broth, 4% [wt/vol] skim milk, and 1.5% [wt/vol] agar). Following incubation at 37°C for 18 h, proteolytically active colonies produced translucent halos due to casein degradation. The extent of proteolytic activity was quantified by measuring the area of clearance zones from high-resolution digital images.

### Pyocyanin quantification

Overnight cultures of *P. aeruginosa*, grown in LB-Mops broth, were diluted in 4 mL of *Pseudomonas P* broth (composed of 20 g/L pancreatic digest of gelatin, 1.4 g/L magnesium chloride, and 10 g/L potassium sulfate) to an initial OD_600_ of ≈0.02. The bacterial cultures were incubated at 37°C for 24 h under aerobic conditions. Following incubation, cells were pelleted by centrifugation at 13,000 × *g* for 3 min. The resulting supernatants were carefully collected, and pyocyanin levels were assessed spectrophotometrically by measuring the absorbance at 695 nm (OD_695_). To normalize pyocyanin production relative to bacterial growth, the ratio of OD_695_ to OD_600_ was calculated and monitored over time.

### Hydrogen cyanide detection assay

HCN production by *P. aeruginosa* strains was assessed using a chromogenic test paper method. The detection system was prepared by soaking Whatman 3MM chromatography paper (cut to agar plate dimensions) in HCN detection reagent containing the following: 5 mg copper (II) ethyl acetoacetate, 5 mg 4,4'-methylenebis(N,N-dimethylaniline), and 1–2 mL chloroform. Overnight LB-MOPS cultures were spot-inoculated onto 2% peptone agar plates and incubated at 37°C for 12 h. The chromogenic test paper was then overlaid on the plates and incubated for an additional 12 h at 37°C. HCN production was indicated by development of a blue coloration on the test paper, which was quantified using ImageJ software (https://imagej.nih.gov/ij).

### Elastase activity assay

Overnight cultures of *P. aeruginosa* strains grown in LB-MOPS broth were diluted to OD_600_ ≈ 0.02 in 4 mL fresh M9-0.5% CAA medium and incubated with shaking at 37°C for 18 h. Following centrifugation to pellet cells, 500 μL of the supernatant was mixed with an equal volume of elastin-Congo Red (ECR) buffer (0.1 M Tris-HCl, 1 mM CaCl₂, 5 mg/mL ECR [Sigma-Aldrich E0502], pH 7.2) and incubated in the dark at 37°C for 2 h. The reaction was terminated by adding 100 μL 0.12 M EDTA and cooling on ice. After centrifugation (5,000 × *g*, 5 min, 4°C) to pellet insoluble ECR, elastase activity was quantified by measuring absorbance at 495 nm of the supernatant.

### Reporter plasmid assays for quorum-sensing systems

To monitor QS system activity, we employed GFP reporter plasmids containing promoter fusions: P*lasI*-GFP for las system expression, P*rhlA*-GFP for rhl system expression, and P*pqsA*-GFP for PQS system expression. These reporter plasmids were introduced into *P. aeruginosa* strains and selected on LB agar plates supplemented with gentamicin (Gm). For assays, PAO1 reporter strains were cultured in LB-MOPS broth with 50 μg/mL Gm for 12 h and then diluted to OD_600_ ≈ 0.02 in fresh Gm-supplemented medium and grown to stationary phase (16 h). Bacterial suspensions were aliquoted (200 μL/well) into 96-well plates with six technical replicates. Fluorescence (excitation: 488 nm; emission: 525 nm) and cell density (OD_600_) were measured using a Synergy H1MF microplate reader (BioTek Instruments).

### Quantification of 3OC_12_-HSL and C4-HSL

*P. aeruginosa* strains were cultured overnight in LB-Mops broth and diluted to a starting OD_600_ ≈ 0.02. Cells were then grown in 4 mL LB at 37°C for 18 h. AHLs were extracted with an equal amount of ethyl acetate. Relative quantification of 3OC_12_-HSL and C4-HSL was performed using a reporter bioassay, in which 3OC_12_-HSL was detected by a Δ*lasI* strain containing P*lasB*-GFP and C4-HSL was detected by a Δ*rhI* strain containing P*rhlA*-GFP.

### Mammalian cell cytotoxicity assay

CHO cells were maintained in RPMI 1640 medium (Gibco, Thermo Fisher Scientific) supplemented with 10% FBS at 37°C under 5% CO₂. For infection assays, exponentially growing *P. aeruginosa* (cultured in LB-MOPS to OD_600_ ≈ 0.5) was harvested by centrifugation (4,000 × *g*, 5 min), washed with PBS, and resuspended in fresh medium. Bacterial suspensions were added to near-confluent CHO monolayers at an MOI of 5:1, followed by incubation at 37°C for 6 h. Cytotoxicity was quantified by measuring LDH release into the supernatant using a commercial LDH Cytotoxicity Detection Kit (Beyotime Biotechnology, Shanghai, China).

### Monoculture experiments

Individual colonies of test strains were inoculated into 3 mL LB-MOPS broth and grown overnight at 37°C. The overnight cultures were then adjusted to an initial OD600 of 0.5, and 100 µL of each suspension was transferred into 3 mL M9-1% casein broth in 14-mL Falcon tubes (Corning, Shanghai, China). Cultures were incubated at 37°C for 18 h, after which phenotypic observations were documented photographically.

### Competition experiments

*P. aeruginosa* strains (wild-type strain and the RpoA289 mutant) grown in LB-Mops broth with shaking at 37℃ for 18 h were diluted to OD_600_ ≈ 2.7. The wild-type strain and the RpoA289 mutant were mixed at a 9:1 ratio. The cultures were diluted at 1:20 into 3 mL M9-1% casein medium in 14-mL Falcon tubes (Corning) and were incubated at 37°C for every 24 h. One hundred microliters of cultures was then spread on LB agar. At least 100 single colonies were spotted on the skim milk agar plates (25% [vol/vol] LB, 4% [wt/vol] skim milk, and 1.5% [wt/vol] agar) to count the number of wild-type (protease-positive) and the RpoA289 mutant (protease-negative) colonies.

### Production and purification of RpoA proteins

The full-length His-tagged RpoA and truncated RpoA289 variant were cloned into the pJN105 expression vector and transformed into *P. aeruginosa* PAO1. Protein expression was induced by culturing in LB broth supplemented with 1 mM L-arabinose at 37°C for 18 h. Cells were harvested by centrifugation (5,000 × *g*, 20 min, 4°C) and resuspended in lysis buffer (PBS containing 1× Protease Inhibitor Cocktail [Bimake, USA] and 1 mg/mL lysozyme). Cell lysis was performed using a high-pressure homogenizer (Stansted Fluid Power Ltd, UK) at 420 MPa, followed by centrifugation (13,000 × *g*, 30 min, 4°C) to clarify the lysate. The supernatant was incubated with Ni-NTA resin (Qiagen, Shanghai, China) at 4°C for 2 h with gentle agitation. The resin was then packed into a chromatography column and sequentially washed with wash buffer (500 mM NaCl, 20 mM NaH₂PO₄, and 5 mM imidazole; pH 8.0). Bound proteins were eluted using elution buffer (500 mM NaCl, 20 mM NaH₂PO₄, and 200 mM imidazole; pH 8.0). Eluted fractions were analyzed by native PAGE, and those containing the target protein were pooled and concentrated using an ultrafiltration device (MilliporeSigma, USA). The final purified protein was buffer-exchanged into PBS and stored at −80°C for downstream applications.

### Electrophoretic mobility shift assay

The promoter regions of *lasI* (P*lasI*) and *lasR* (P*lasR*) genes were utilized as probes for EMSA. Probe DNA was biotin-labeled following our established protocol (49), using biotin-11-UTP (Jena Bioscience, Germany) and T4 DNA polymerase (Novoprotein, China) during PCR amplification. For binding reactions, 30 ng of the labeled probe was incubated with 0.3–1.5 µg of purified His-tagged RpoA or RpoA289 protein in binding buffer (10 mM Tris-HCl, 50 mM KCl, 1 mM DTT, 1% [vol/vol] glycerol, 10 mM MgCl₂, and 1 µg/µL poly(dI-dC); pH 7.5) at 25°C for 30 min. DNA-protein complexes were resolved on a 5% polyacrylamide gel in 0.5× TBE buffer under electrophoresis conditions of 90 V for 30 min, followed by 100 V for 150 min. Resolved DNA was transferred to a nylon membrane and crosslinked using a UV Crosslinker (UVP CX-2000, USA; 1200 J/cm² for 60 s). The membrane was blocked with 5% (wt/vol) nonfat milk in maleic acid buffer (100 mM maleic acid and 150 mM NaCl; pH 7.5) for 1 h at room temperature, followed by incubation with streptavidin-HRP conjugate (Thermo Fisher Scientific) in 1% milk-blocking buffer for 1 h. After 4 5-min washes with TBST (50 mM Tris, 150 mM NaCl, and 0.05% Tween-20; pH 7.5), DNA-protein interactions were detected using the Immobilon Western Chemiluminescent HRP Substrate (Millipore) and imaged on a Tanon 5200 Multi System (Tanon, Shanghai, China).

### Whole-genome sequencing

Microbial genomic DNA (1 μg) was sheared to an average fragment size of ~350 bp using a Covaris S220 ultrasonicator (Covaris, Woburn, MA, USA). An Illumina-compatible DNA library was prepared using the Next-Generation Sequencing DNA Library Preparation Kit (Novagen, Tianjin, China) following the manufacturer’s protocol. Briefly, fragmented DNA underwent end repair and adapter ligation, followed by purification with AMPure XP beads (Agencourt Beckman Coulter, USA). The adapter-ligated products were then PCR-amplified using adapter-specific primers and subsequently purified again with AMPure XP beads. Finally, the library was subjected to paired-end sequencing (PE150) on an Illumina NovaSeq 6000 platform.

### Analysis of Illumina HiSeq short reads

Raw short-read sequencing data underwent stringent quality control, including adapter trimming using Cutadapt (Novagen, Tianjin, China), to generate high-quality reads. These processed reads were then aligned to the *P. aeruginosa* PAO1 reference genome (NCBI accession: NC_002516.2) using the Burrows-Wheeler Aligner (BWA). Genome-wide variant calling was subsequently performed on the aligned reads using SAMtools (50) supplemented with custom Perl scripts for enhanced detection.

### Multiple sequence alignment

Homologous RpoA protein sequences from diverse bacterial species, as detailed in Table S7, were aligned using the Clustal Omega (https://www.ebi.ac.uk/Tools/msa/clustalo/) tool for multiple sequence alignment.

### Protein structural modeling

The *P. aeruginosa* RpoA structure was determined using the AlphaFold Protein Structure Database (ID: O52760) (https://alphafold.ebi.ac.uk/entry/O52760). The mutated residues of RpoA were visualized by the PyMOL molecular graphics system (version 2.5.2).

### Software

The following software was used in this study: BWA software, version 0.7.15-r1140 (http://bio-bwa.sourceforge.net) (51); Cutadapt software, version 1.16 (https://github.com/marcelm/cutadapt/blob/main/doc/guide.rst) (52); Samtools software, version 1.5 (http://samtools.sourceforge.net) (50); FastQC, version fastqc_v0.11.5 (https://www.bioinformatics.babraham.ac.uk/projects/fastqc/); Hisat2, version 2.1.0 (https://daehwankimlab.github.io/hisat2/) (53); HTSeq, version 0.11.1 (https://htseq.readthedocs.io/en/release_0.11.1/count.html) (54); DESeq2 (55); Perl software, version v5.22.1 (https://www.perl.org/); Python software, version v3.8.2 (https://www.python.org/); R software, version v3.6.1 (http://www.R-project.org/); GraphPad Prism software, version 5 (https://www.graphpad.com/); and PyMOL, version 2.6 (http://www.pymol.org/).

### Statistical analysis

A biological replicate constitutes a repetition of a given experiment by measurement using biologically distinct samples, such as distinct individual isolates. Statistical analysis was performed using Excel, GraphPad Prism, and R software (http://www.R-project.org/).

## Data Availability

The sequencing data sets generated in this study have been deposited in the NCBI SRA database (https://www.ncbi.nlm.nih.gov/sra) and are accessible under the Bioproject accession number PRJNA1304742.

## SUPPLEMENTAL MATERIAL

Supplemental material mbio.00032-26-s0001.docx Supplemental figures and captions for supplemental tables.

Table S1 mbio.00032-26-s0002.xlsx Read mapping statistics of whole-genome re-sequencing data.

Table S2 mbio.00032-26-s0003.xlsx Identification of mutations in protease-negative colonies derived from the engineered strain with an extra copy of *lasR* and *groEL*.

Table S3 mbio.00032-26-s0004.xlsx Comparison of secondary structures between wild-type RpoA and the RpoA289 variant.

Table S4 mbio.00032-26-s0005.xlsx List of *P. aeruginosa* RpoA variant strains.

Table S5 mbio.00032-26-s0006.xlsx Strains and plasmids used in this study.

Table S6 mbio.00032-26-s0007.xlsx Oligonucleotides used in this study.

Table S7 mbio.00032-26-s0008.xlsx RpoA homolog sequences used in the study.

Table S8 mbio.00032-26-s0009.xlsx RpoA variants tested in this study

Table S9 mbio.00032-26-s0010.xlsx Source data and statistic analysis.

ASM does not own the copyrights to Supplemental Material that may be linked to, or accessed through, an article. The authors have granted ASM a non-exclusive, world-wide license to publish the Supplemental Material files. Please contact the corresponding author directly for reuse.
