# Supplementary material for: Novel dual regulatory roles of RpoA in quorum sensing regulation and social behavior switching in Pseudomonas aeruginosa
Source: mBio. 2026 Mar 24;17(5):e00032-26. doi: 10.1128/mbio.00032-26 (PMC13170360; doi:10.1128/mbio.00032-26)
Supplement: Supplemental material — Supplemental figures and captions for supplemental tables. [file mbio.00032-26-s0001.docx]

**Supplementary Information**

Novel dual regulatory roles of RpoA in quorum sensing regulation and social behavior switching in *Pseudomonas aeruginosa*

Huali Chen^1^, Yonglin Liang^1^, Xiaoqing Zhou^1^, Wenjie Cai^1^^,2^, Huifang Qiu^1^, Ajai A. Dandekar^3^, Weijun Dai^1^*

**Supplementary figures**

**Figure S1.** Whole-genome sequencing (WGS) analysis of the evolved mutant.

**Figure S2.** Complementation of RpoA-deficient mutants with wild-type *rpoA* restores QS-controlled proteolytic activity.

**Figure S3.** RpoA modulates QS in a LasR-dependent manner.

**Figure S4.** Amino acid substitution in the RpoA289 variant induces conformational changes.

**Figure S5.** Reduced transcriptional expression of *lasI* and *lasR* genes in the RpoA289 mutant.

**Figure S6.** Amino acid substitution frequency profile of RpoA variants in natural *P. aeruginosa* isolates.

**Figure S7.** Production of QS-controlled metabolites in RpoA variant strains.

**Figure S8.** Comparative sequence alignment of RpoA homologs across different bacterial species.

**Figure S9.** Two putative functionally distinct clusters within the α-CTD of RpoA.

**Supplementary tables**

**Table S1.** Read mapping statistics of whole-genome re-sequencing data.

**Table S2.** Identification of mutations in protease-negative colonies derived from the engineered strain with an extra copy of *lasR* and *groEL*.

**Table S3.** Comparison of secondary structures between wild-type RpoA and the RpoA289 variant.

**Table S4.** List of *P. aeruginosa* RpoA variant strains.

**Table S5.** Strains and plasmids used in this study.

**Table S6.** Oligonucleotides used in this study.

**Table S7.** RpoA homolog sequences used in the study.

**Table S8.** RpoA variants tested in this study.

**Table S9.** Source data and statistic analysis.


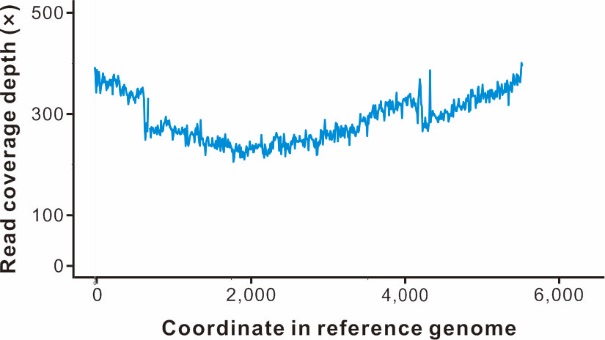


**Figure S1. Whole-genome sequencing (WGS) analysis of the evolved mutant.** Read coverage depth of whole genome sequencing of the evolved mutant. Average fold-coverages within a 10 kb window are plotted against the *P. aeruginosa* PAO1 reference genome.

**
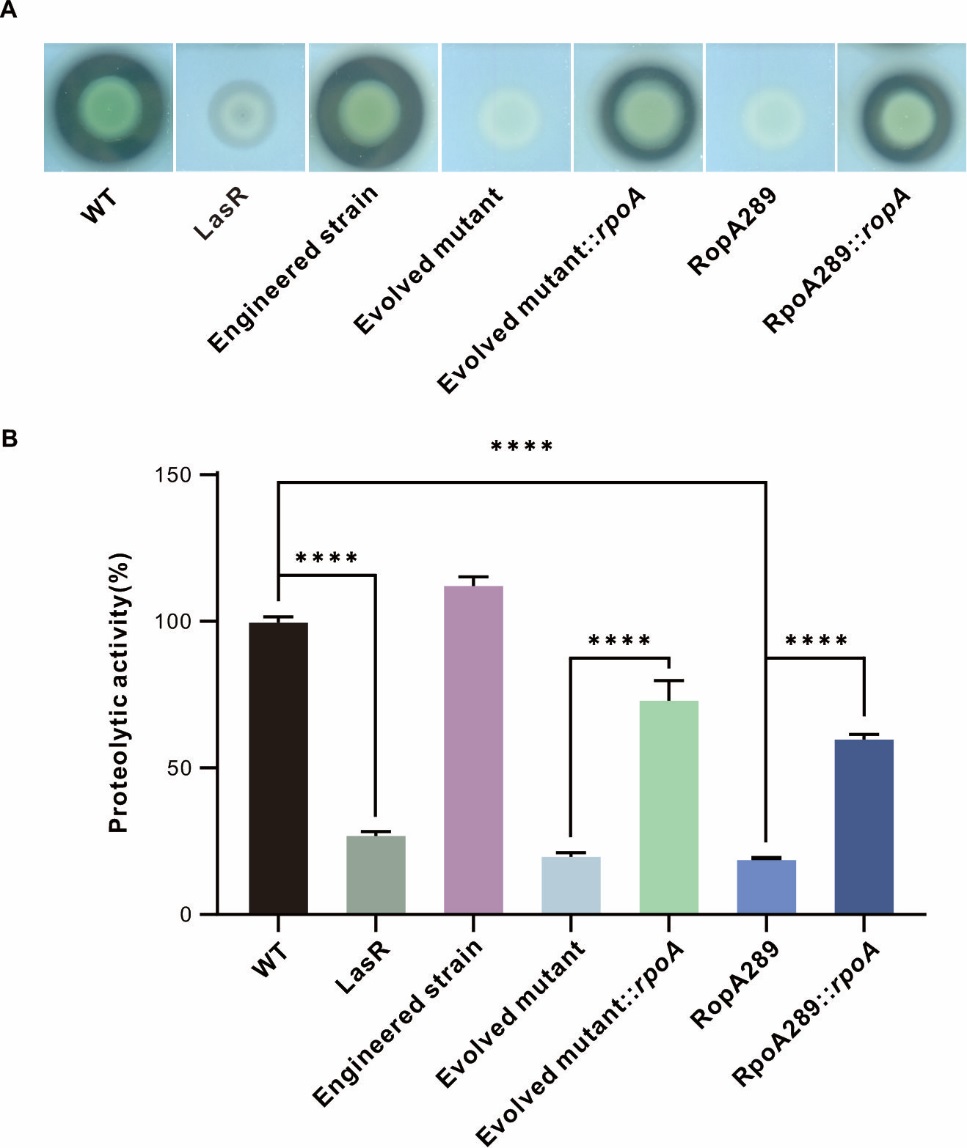
**

**Figure S2. Complementation of RpoA-deficient mutants with wild-type rpoA restores QS-controlled proteolytic activity.**  The introduction of an episomal copy of the wild-type *rpoA* gene into the RpoA-deficient mutants rescued the proteolytic activity regulated by QS. (A) Skim milk agar plate assay demonstrating proteolytic activity. Equal amounts of bacterial cultures were spotted onto skim milk agar plates and incubated at 37oC for 24 h. Clear zones around colonies indicate protease activity. (B) Quantification of proteolytic activity based on the assay in (A), analyzed using ImageJ software. Data are present means ± standard deviation (SD) (*n* = 3). A one-way ANOVA with a Bonferroni post hoc test was used to compare the groups (**P* < 0.05, ***P* < 0.01, ****P* < 0.001). WT, wild-type PAO1 strain; LasR, LasR-null mutant; Engineered strain, the PAO1 strain containing a miniTn7-*lasR-groE*L construct; Evolved mutant, the engineered strain evolved in casein medium and the protease-negative mutant was screened; Evolved mutant::*rpoA*, the evolved mutant integrated with a miniTn7-PrrnB-*rpoA* construct; RpoA289, the RpoA289 variant mutant; RpoA289:*:rpoA*, the RpoA289 variant mutant integrated with a miniTn7-PrrnB-*rpoA* construct.

**
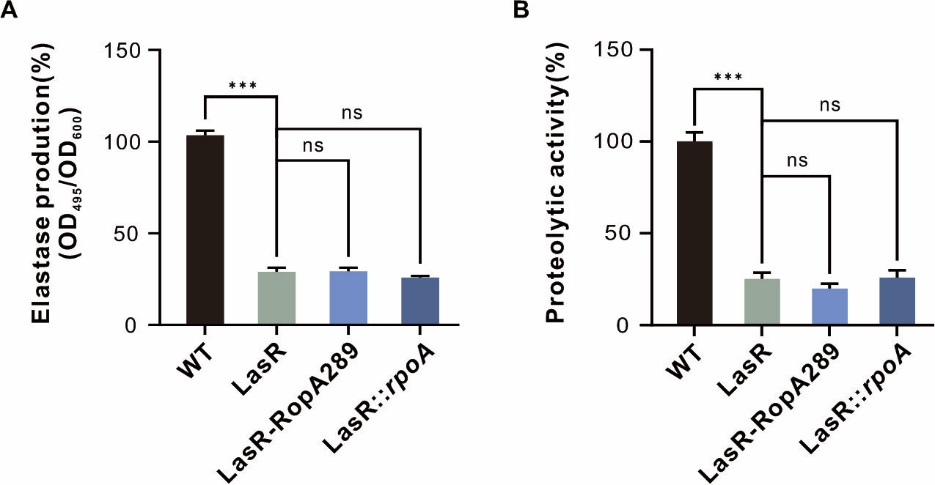
**

**Figure S3. RpoA modulates QS in a LasR-dependent manner.** (A) Elastase activity, measured as OD_495_/OD_600_ values, in the indicated strains. (B) Proteolytic activity assessed by a kim milk agar plate assay. Equal volums of bacterial cultures were spotted onto plates and incubated at 37oC for 24 h. Quantification of proteolytic halo areas using ImageJ software. Data present the means ± standard deviation (SD) (*n* = 3). Statistical significance was determined by one-way ANOVA with Bonferroni’s post hoc test (**P* < 0.05, ***P* < 0.01, ****P* < 0.001). WT, wild-type PAO1 strain; LasR, LasR-null mutant; LasR-RpoA289, the LasR-null mutant containing a RpoA289 variant; LasR::*rpoA*, the LasR-null mutant integrated with a miniTn7-PrrnB-*rpoA* construct.

**
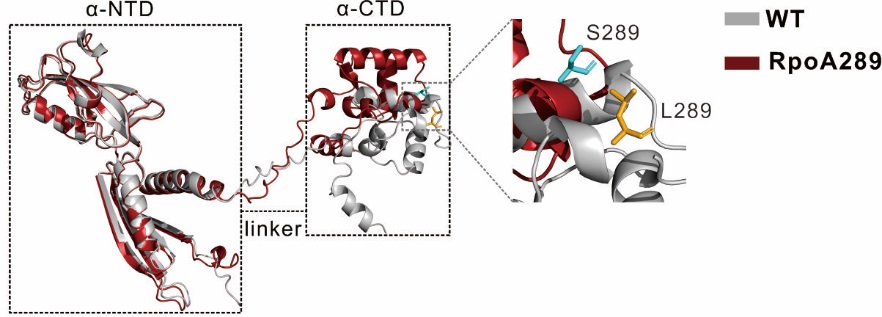
**

**Figure S4. Amino acid substitution in the RpoA289 variant induces conformational changes.** Three-dimensional structure of wild-type (WT) RpoA and the RpoA289 variant were predicted by AlphaFold 3. Structural alignment and visualization were performed with PyMOL software. A magnified view highlights the original residue (S289) and its substitution (L289) in the α-CTD region.

**
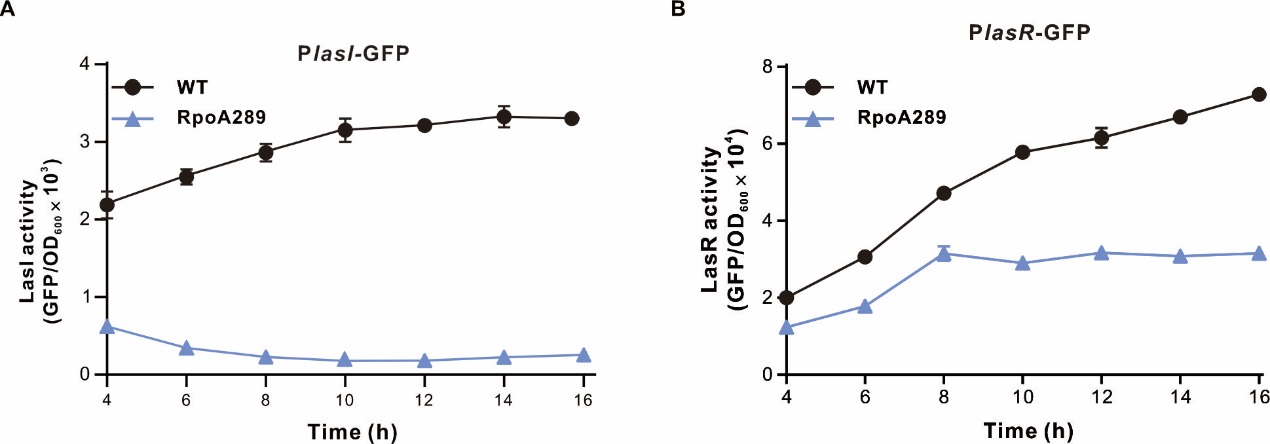
**

**Figure S5. Reduced transcriptional expression of *lasI* and *lasR* genes in the RpoA289 mutant.** (A-B) Transcriptional from the *lasI* (A) and *lasR* (B) promoters was assessed using P*lasI*-GFP and P*lasR*-GFP reporter fusions, respectively. Data represented as means ±standard deviation (SD) of at least three independent experiments ( *n* ≧ 3).


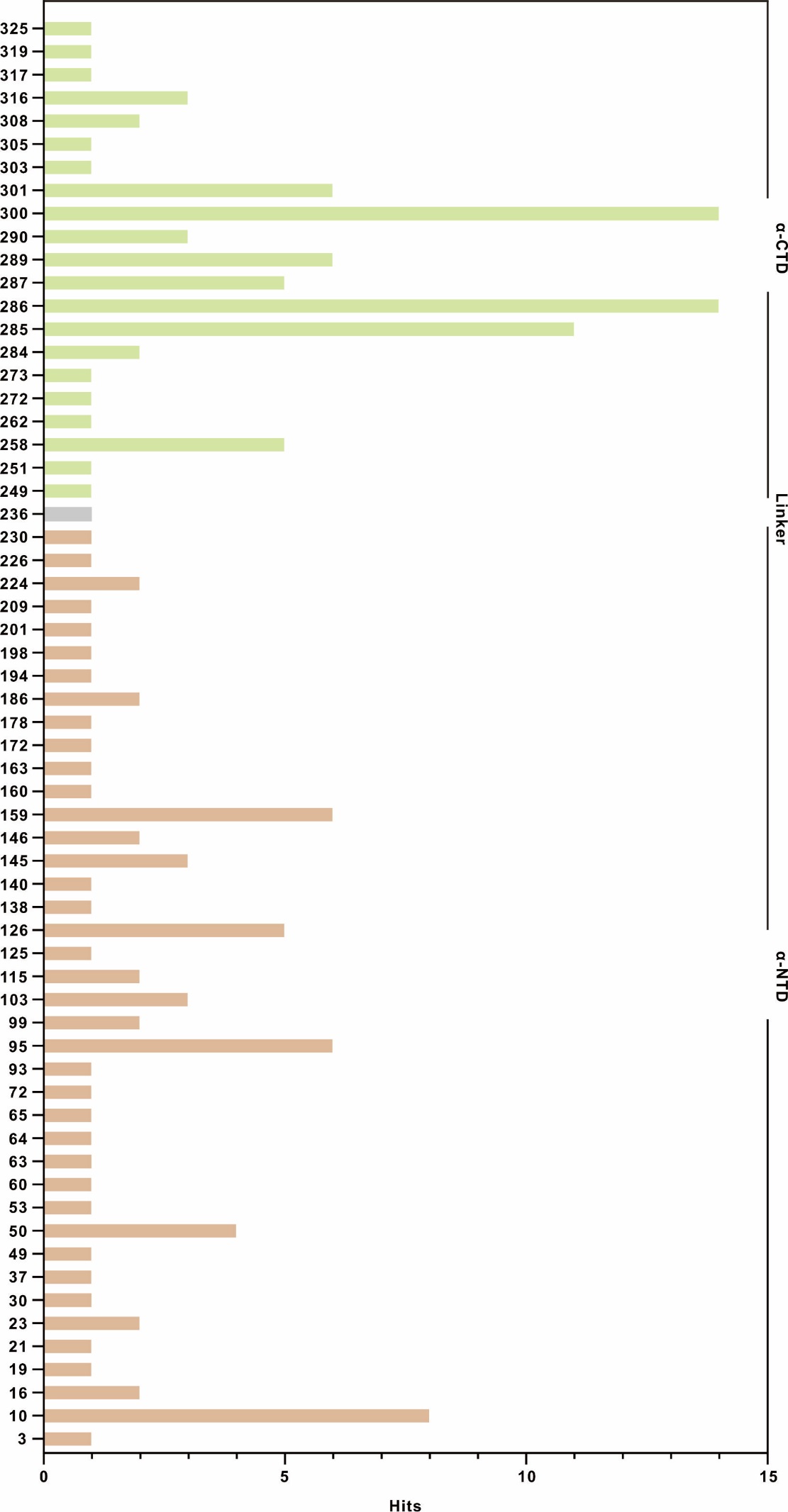


**Figure S6. Amino acid substitution frequency profile of RpoA variants in natural P. aeruginosa isolates.** The histogram displays the occurrence frequency of amino acid substitutions across the RpoA sequence, as identified in natural *P. aeruginosa* isolates. Each bar represents the number of independent substitution events detected at specific residue positions.

**
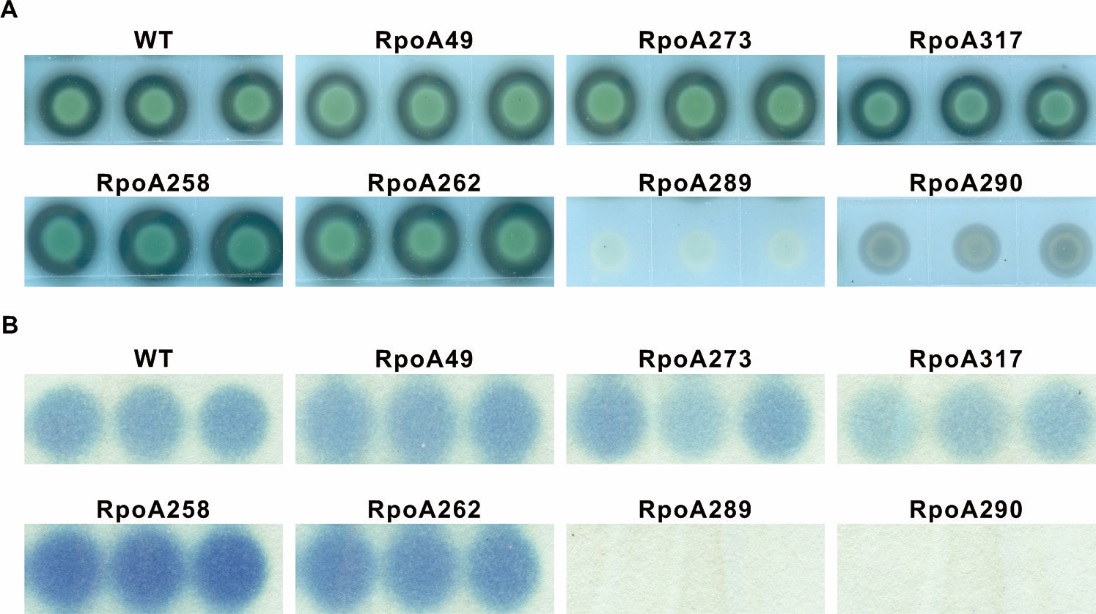
**

**Figure S7. Production of QS-controlled metabolites in RpoA variant strains.** (A) Proteolytic activity as visualized on the skim milk plate. Equal amounts of bacterial cultures were spotted onto plates and incubated at 37oC for 24 hours, with proteolysis measured by zones of clearing around colonies. (B) Cyanide production was measured using cyanide-sensitive filter papers after growth in 6-well plates at 37oC for 24 hours. The experiment was repeated three times with similar results.

**
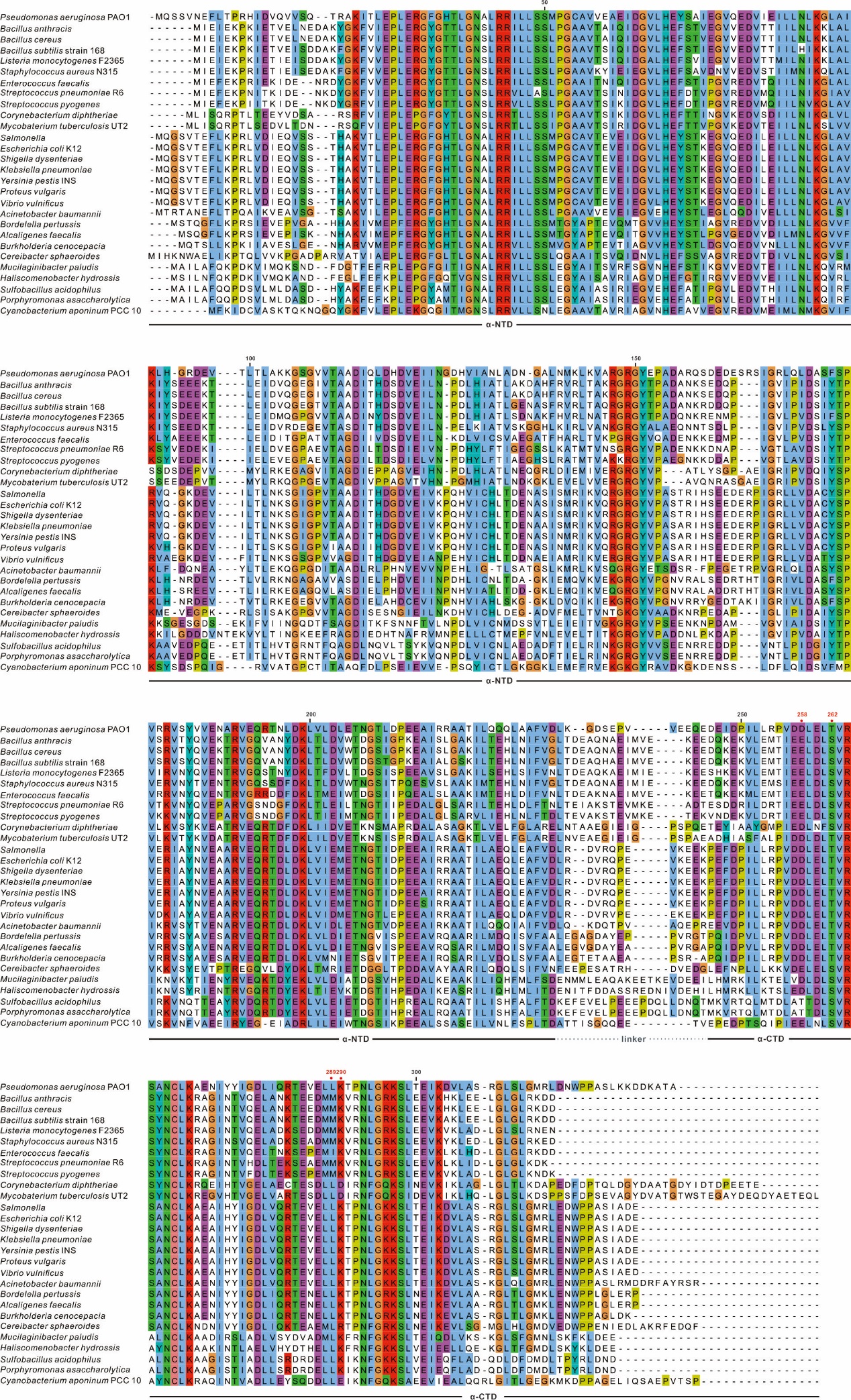
**

**Figure S8. Comparative sequence alignment of RpoA homologs across different bacterial species.** Multiple sequence alignment of RpoA proteins from diverse bacterial species was conducted using Clustal Omega. Conserved amino acids are highlighted based on their degree of evolutionary conservation. The amino acid substitutions in QS-related RpoA variants are specially marked in red for emphasis.

**
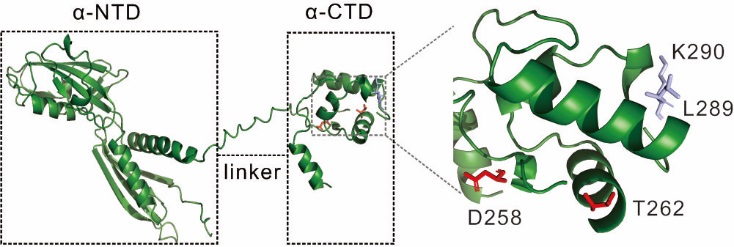
**

**Figure S9. Two putative functionally distinct clusters within the α-CTD of RpoA.** Three-dimensional structure of wild-type RpoA modelled by AlphaFold3, highlighting the key residues (D258, T262, L289 and D290) involved in QS regulation. Magnified view showing the spatial organization of these residues into two putative functional clusters. Structural visualization was performed using PyMOL software.
